# Italian real life experience with ibrutinib: results of a large observational study on 77 relapsed/refractory mantle cell lymphoma

**DOI:** 10.18632/oncotarget.25215

**Published:** 2018-05-04

**Authors:** Alessandro Broccoli, Beatrice Casadei, Alice Morigi, Federico Sottotetti, Manuel Gotti, Michele Spina, Stefano Volpetti, Simone Ferrero, Francesco Spina, Francesco Pisani, Michele Merli, Carlo Visco, Rossella Paolini, Vittorio Ruggero Zilioli, Luca Baldini, Nicola Di Renzo, Patrizia Tosi, Nicola Cascavilla, Stefano Molica, Fiorella Ilariucci, Gian Matteo Rigolin, Francesco D'Alò, Anna Vanazzi, Elisa Santambrogio, Roberto Marasca, Lucia Mastrullo, Claudia Castellino, Giovanni Desabbata, Ilaria Scortechini, Livio Trentin, Lucia Morello, Lisa Argnani, Pier Luigi Zinzani

**Affiliations:** ^1^ Institute of Hematology "L. e A. Seràgnoli", University of Bologna, Bologna, Italy; ^2^ Operative Unit of Medical Oncology, IRCCS Fondazione Maugeri, Pavia, Italy; ^3^ Department of Hematology Oncology, Fondazione IRCCS Policlinico San Matteo, Pavia, Italy; ^4^ Division of Medical Oncology A, National Cancer Institute, Aviano, Italy; ^5^ Department of Hematology, DISM, Azienda Sanitaria Universitaria Integrata, Udine, Italy; ^6^ Division of Hematology, Department of Molecular Biotechnologies and Scienze for Health, University Torino, Torino, Italy; ^7^ Unit of Hematology, Fondazione IRCCS Istituto Nazionale dei Tumori, Milano, Italy; ^8^ Hematology and Transplantation Unit, Regina Elena National Cancer Institute, Roma, Italy; ^9^ Unit of Hematology, Ospedale di Circolo, Fondazione Macchi, Varese, Italy; ^10^ Department of Cell Therapy and Hematology, San Bortolo Hospital, Vicenza, Italy; ^11^ Hematology Service, Medicine Department, Rovigo Hospital, Rovigo, Italy; ^12^ Division of Hematology, Niguarda Ca’ Granda Hospital, Milano, Italy; ^13^ OncoHematology Unit, Fondazione Ca' Granda IRCCS Ospedale Maggiore Policlinico, Milano, Italy; ^14^ Unit of Hematology, Vito Fazzi Hospital, Lecce, Italy; ^15^ Hematology Unit, Infermi Hospital Rimini, Rimini, Italy; ^16^ IRCCS, Casa Sollievo della Sofferenza, San Giovanni Rotondo, Italy; ^17^ Unit of Oncology/Hematology, Azienda Ospedaliera "Pugliese-Ciaccio", Catanzaro, Italy; ^18^ Unit of Hematology, Arcispedale Santa Maria Nuova di Reggio Emilia, Reggio Emilia, Italy; ^19^ Unit of Hematology, Azienda Ospedaliero-Universitaria di Ferrara, Ferrara, Italy; ^20^ Institute of Hematology, Università Cattolica del Sacro Cuore, Roma, Italy; ^21^ Hemato-Oncology Division, European Institute of Oncology, Milano, Italy; ^22^ Unit of Hematology, University-Hospital Città della Salute e della Scienza di Torino, Torino, Italy; ^23^ Department of Medical Sciences, Hematology Unit, University of Modena and Reggio Emilia, Modena, Italy; ^24^ Unit of Hematology, Ospedale San Gennaro di Napoli, Napoli, Italy; ^25^ Unit of Hematology, Ospedale Santa Croce E Carle, Cuneo, Italy; ^26^ Ematologia Clinica, Ospedale Maggiore, Trieste, Italy; ^27^ Clinica di Ematologia Ospedali Riuniti, Ancona, Italy; ^28^ Unit of Hematology, University of Padova, Padova, Italy; ^29^ Humanitas Cancer Center, Istituto Clinico Humanitas, Rozzano, Italy

**Keywords:** ibrutinib, mantle cell lymphoma, relapsed, refractory, real life

## Abstract

Although sometimes presenting as an indolent lymphoma, mantle cell lymphoma (MCL) is an aggressive disease, hardly curable with standard chemo-immunotherapy. Current approaches have greatly improved patients’ outcomes, nevertheless the disease is still characterized by high relapse rates. Before approval by EMA, Italian patients with relapsed/refractory MCL were granted ibrutinib early access through a Named Patient Program (NPP).

An observational, retrospective, multicenter study was conducted. Seventy-seven heavily pretreated patients were enrolled. At the end of therapy there were 14 complete responses and 14 partial responses, leading to an overall response rate of 36.4%. At 40 months overall survival was 37.8% and progression free survival was 30%; disease free survival was 78.6% at 4 years: 11/14 patients are in continuous complete response with a median of 36 months of follow up. Hematological toxicities were manageable, and main extra-hematological toxicities were diarrhea (9.4%) and lung infections (9.0%). Overall, 4 (5.2%) atrial fibrillations and 3 (3.9%) hemorrhagic syndromes occurred.

In conclusions, thrombocytopenia, diarrhea and lung infections are the relevant adverse events to be clinically focused on; regarding effectiveness, ibrutinib is confirmed to be a valid option for refractory/relapsed MCL also in a clinical setting mimicking the real world.

## INTRODUCTION

Mantle cell lymphoma (MCL) is a rare, clinically aggressive B-cell lymphoma that accounts for 6–8% of all non-Hodgkin lymphoma cases [1]. It is a disease that predominantly affects older men (median age, 65 years), usually presents as late-stage disease and is associated with a poor prognosis [2]. While high response rates are seen with induction chemo-immunotherapy, relapse is almost universal, occurring linearly even beyond 6 years from the end of therapy [3]. Management of relapsed disease is challenging, and treatment decisions are based on patient's and disease characteristics. Following progression, subsequent treatment is often ineffective and survival is short [4].

Ibrutinib is an oral inhibitor of B-cell receptor signaling through targeting the Bruton's tyrosine kinase (BTK) which has become the preferred therapy at relapse for a majority of patients. In the landmark phase II international trial reported by Wang *et al*., 111 patients with relapsed/refractory MCL (rrMCL) were treated with ibrutinib 560 mg daily until disease progression or unacceptable toxicity [5]. An ORR of 68% was achieved including 21% complete responses (CR). With extended follow-up, median progression-free survival (PFS) was 13 months with median overall survival (OS) of 22.5 months [6]. The median duration of response (DoR) was 17.5 months with 31% PFS at 24 months. Patients with refractory rather than relapsed disease at study entry had inferior outcomes with a median OS of 13 months. Ibrutinib was compared with temsirolimus in a phase III trial of 280 patients with rrMCL. Ibrutinib was associated with a greater ORR (72%, *P* < 0.0001) and CR rate (19%) as well as a significantly longer PFS (14.6 vs. 6.2 months, *P* < 0.0001) [2]. After ibrutinib approval by FDA and before official approval by EMA, patients with rrMCL with unsatisfied critical medical urgency were granted ibrutinib early access through a Named Patient Program (NPP) by compassionate use in Italy. Herein, we report the Italian multicenter experience with ibrutinib in rrMCL as we believe that data collected outside a controlled trial give useful additional information about the clinical use, effectiveness, and safety profile of the drug when applied in a real life context.

## RESULTS

Thirty-three Centers were initially involved, 29 Centers were actually activated. Of the 80 patients expected, 77 were actually enrolled (3.7% difference).

Characteristics of the 77 patients are summarized in Table 1. Participants had an Eastern Cooperative Oncology Group performance status score ≤2, and normal organ function including peripheral blood counts within the normal range. All patients underwent baseline assessments including physical examination, routine hematology and biochemistry as well as imaging prior to therapy. Patients received ibrutinib at the initial dose of 560 mg daily. First diagnosis of MCL was established between 1995 and 2014. The median age at ibrutinib was 65.2 years (range, 34.6–81.3 years); fifty-nine patients were males and 18 were females. Fourteen (18.2%) had systemic symptoms at baseline; an advanced stage (i.e. stage III or IV) was present in 69 (89.6%) patients.

**Table 1:** Patient demographics and characteristics at baseline

	Total population
Patients, *N*	77
Median age at diagnosis, years (range)	65.2 (34.6–81.3)
Median time from diagnosis-ibrutinib, years (range)	68.6 (38.5–83.7)
Median time from last relapse-ibrutinib, days (range)	37 (10–360)
Males, *N* (%)	59 (76.6)
Females, *N* (%)	18 (23.4)
Previous cardiac problems, *N* (%)	2 (2.6)
Stage at diagnosis, *N* (%)	
- I/II (E^*^)	4 (5.2)
- III	12 (15.6)
- IV	61 (79.2)
Stage at ibrutinib, *N* (%)	
- I/II	8 (10.4)
- III	14 (18.2)
- IV	55 (71.4)
Blastoid variant, *N* (%)	3 (3.9)
ECOG^†^ performance status, *N* (%)	
- 0	37 (48.1)
- 1	24 (31.2)
- 2	15 (19.5)
- 3	1 (1.2)
B symptoms, *N* (%)	14 (18.2)
Last therapy before ibrutinib, *N* (%)	
RCHOP^a^	17 (22.1)
Bendamustine	19 (24.7)
Lenalidomide	20 (26.0)
Temsirolimus	4 (5.2)
Bortezomib	6 (7.8)
RBAC^b^	9 (11.7)
transplant	2 (2.6)
- Refractory to most recent therapy, *N* (%)	17 (22.1)
- Refractory to first line therapy, *N* (%)	37 (48.1)

Abbreviations: ^*^E: extranodal; ^†^ECOG: Eastern Cooperative Oncology Group; ^a^RCHOP: rituximab, cyclophosphamide, doxorubicin, vincristine, and prednisone; ^b^RBAC: rituximab, bendamustine and cytarabine.

The median number of prior lymphoma-related systemic regimens was 3 (range, 1–10) including high dose chemotherapy and autologous stem cell transplantation (ASCT) in 27 (35%) patients. Twenty-one (27.3%) had already received bortezomib, 8 (10.4%) temsirolimus, and 25 (32.5%) lenalidomide. Thirty-seven (48.1%) patients had a disease that was refractory to frontline therapy (primary refractory patients) and 17 patients (22.1%) had a disease that was refractory to last therapy before ibrutinib.

### Response

All the patients received ibrutinib for a median of 6 cycles (range, 1–20). Among the 77 patients, 14 (18.2%) achieved CR and 14 (18.2%) obtained a PR with an ORR of 36.4%; among the remaining patients, 8 (10.4%) had stable disease (SD) and 41 (53.3%) showed progression of disease (PD), respectively.

Among the 37 primary refractory patients, 3 (8.1%) achieved CR and 2 (5.4%) had a PR yielding an ORR of 13.5%; in the subset of the 17 patients who were refractory to the last line we observed 4 (23.5%) CR and 3 (17.6%) PR, with an ORR of 50.1%. The difference in ORR between these two subsets of patients is statistically significant (*p* < 0.05). No differences in outcome were observed between patients refractory to and patients relapsed after last therapy before ibrutinib. The number of previous therapies does not affect patients’ responses and outcomes.

**Figure 1:**
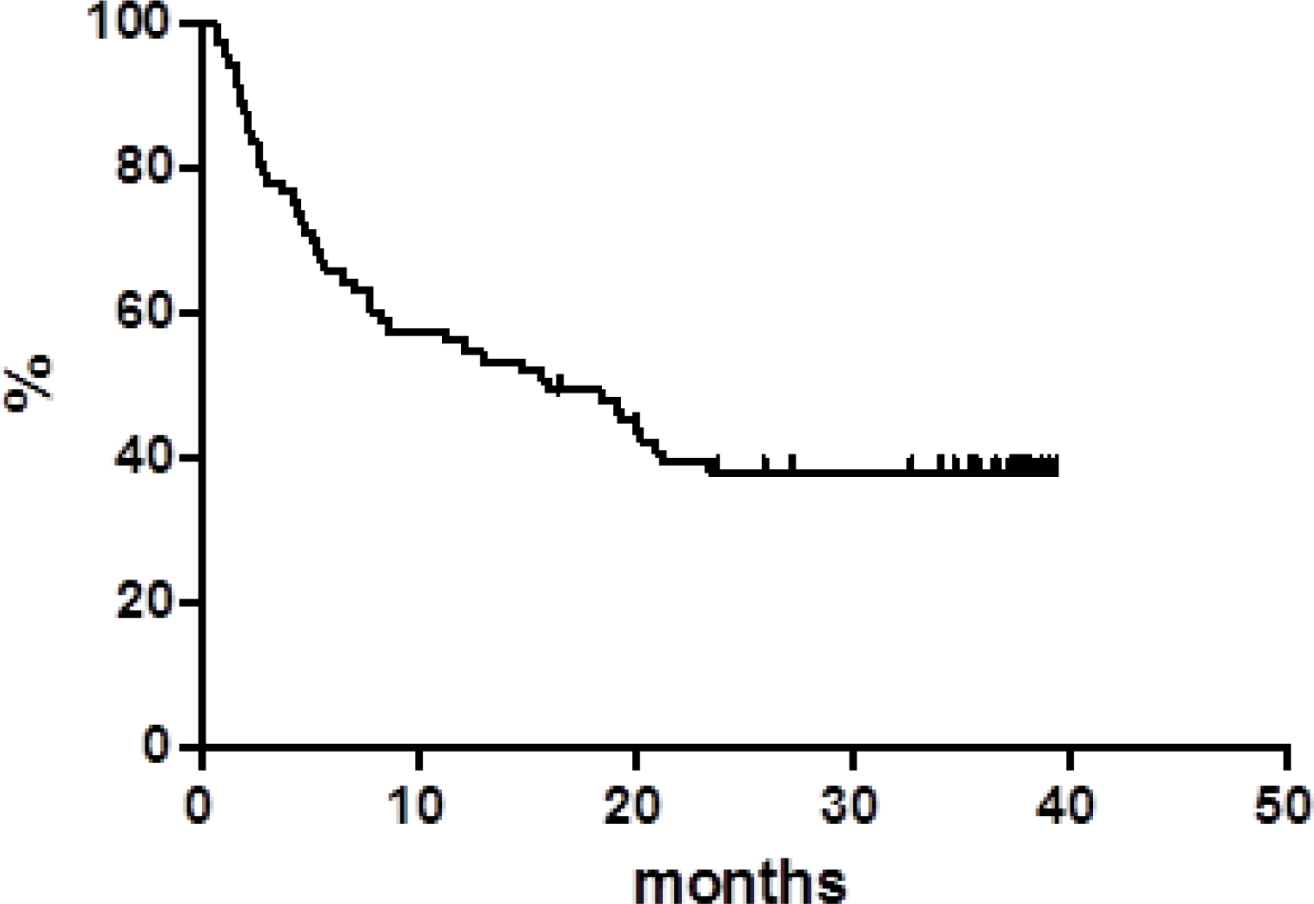
Overall survival

**Figure 2:**
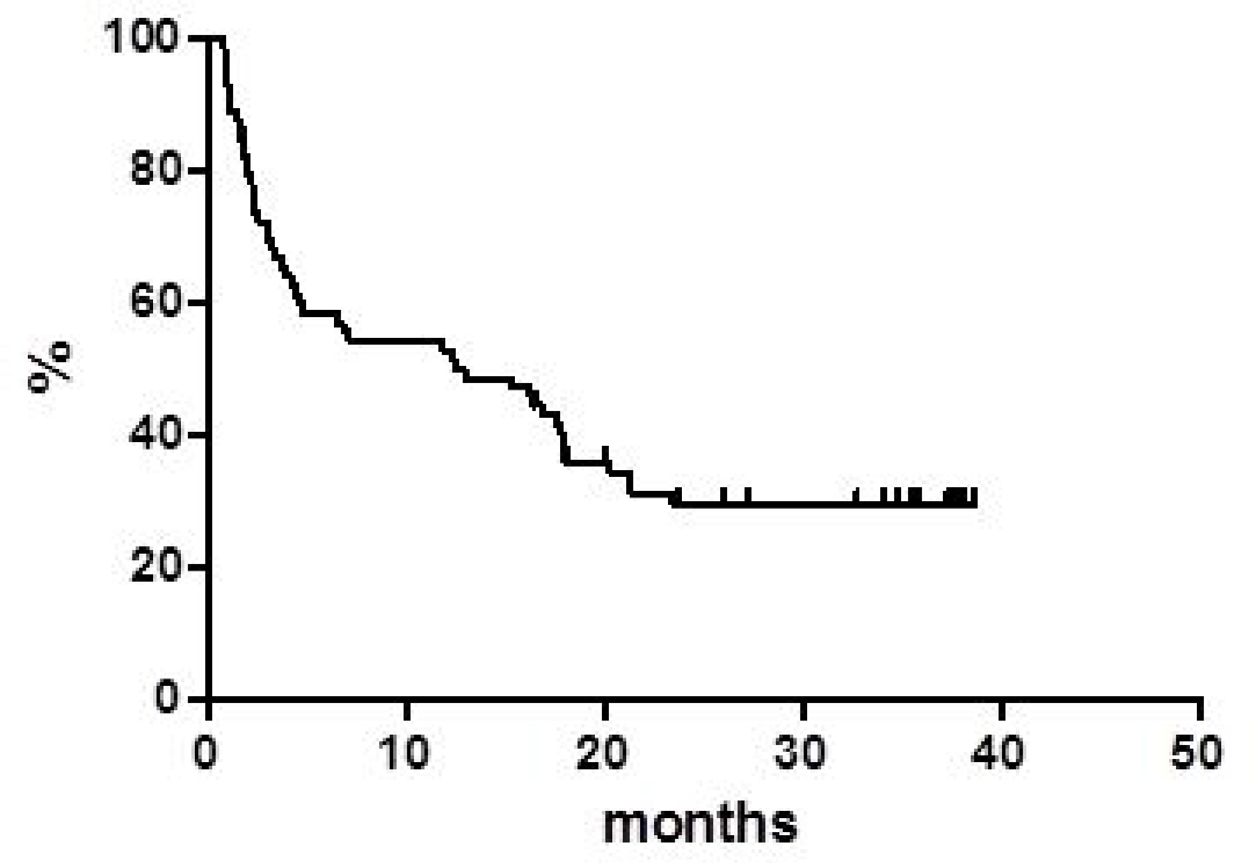
Progression free survival

**Figure 3:**
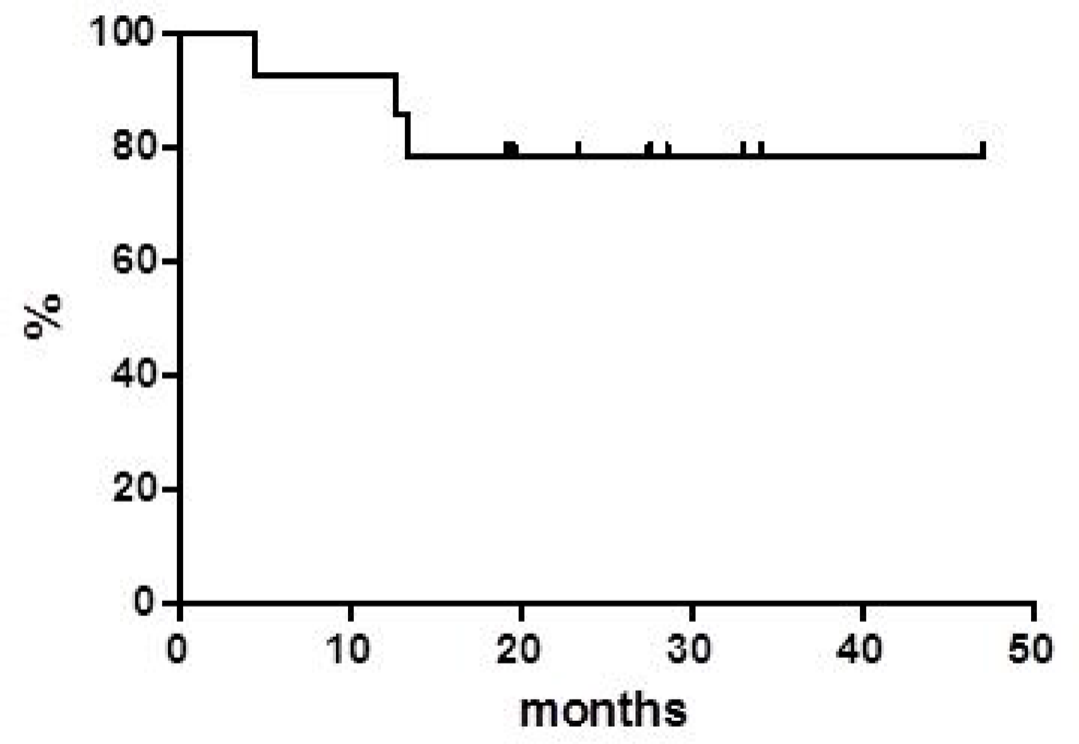
Disease free survival

**Figure 4:**
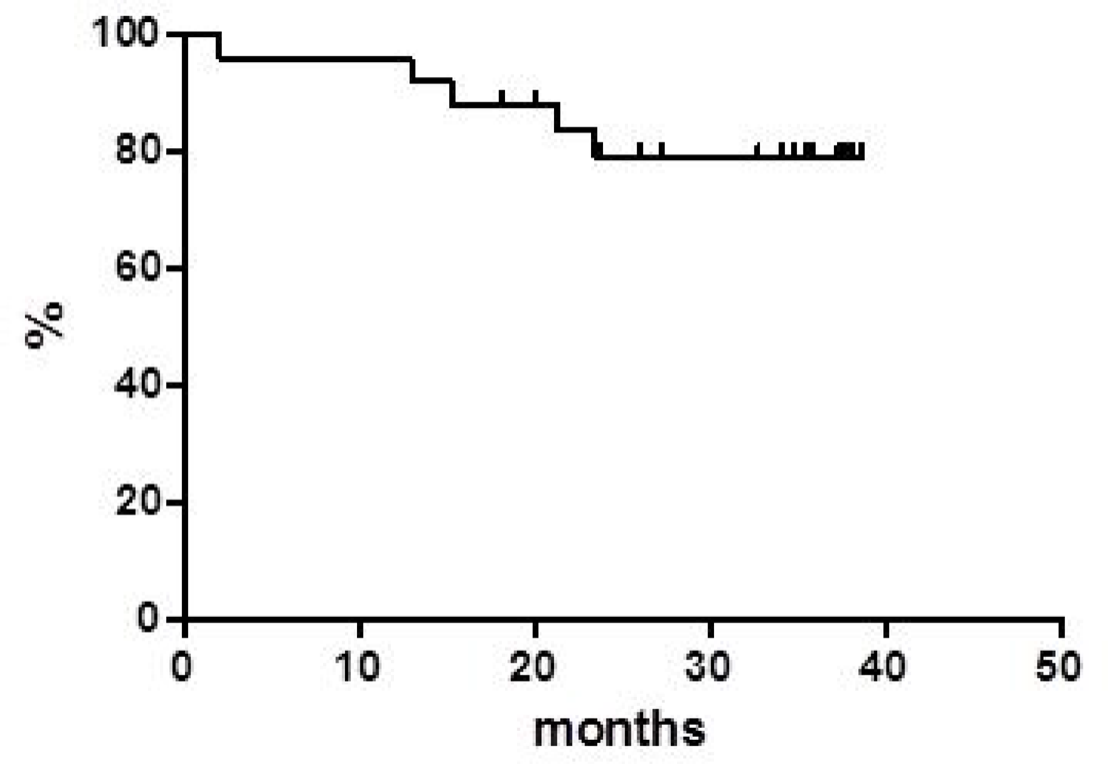
Duration of response

Globally, at a median follow up of 38 months, OS was 37.8% at 40 months (Figure 1) with a median of 16 months. PFS at 40 months was 30% and the median was reached at 12.9 months (Figure 2). Disease free survival (DFS) was 78.6% at 48 months (Figure 3) since 3 (21.4%) out of 14 CR patients relapsed while 11 patients were in continuous CR (CCR) at the latest follow up, with a median DoR of 36 months; among these patients, three received a transplant consolidation (1 with ASCT and 2 with allogeneic transplant [alloSCT]). The DoR was 79.2% at 40 months (Figure 4).

Globally, 37 patients underwent further treatments after ibrutinib; among them, 32 patients received subsequent chemotherapy or chemoimmunotherapy, including 5 patients who received lenalidomide, one temsirolimus, one bortezomib; 7 were rechangelled with ibrutinib. The remaining 5 patients received ibrutinib as bridge to transplant. In particular: 1 PR patient remained in PR after alloSCT; 1 PR converted to a CR with alloSCT; 2 patients in CR consolidated their response with alloSCT and ASCT respectively; 1 patient in CR rapidly developed an acute myeloid leukemia (AML) after alloSCT. Seven patients were rechallenged with ibrutinib: 2 achieved a CR, while the other 5 acquired resistance to drug.

### Safety

Besides PD, reasons for early discontinuations (*N* = 19) were: transplant procedure (*N* = 3), 1 lung cancer, 1 AML, 1 death due to unknown cause, and adverse event (AE) (*N* = 13). AEs in detail were: 2 diarrhea, 1 atrial fibrillation, 2 infections, 1 herpes zoster, 2 hemorrhages, 5 leukocytosis/lymphocytosis. The patient with atrial fibrillation did not interrupt ibrutinib at the onset of arrhythmia: he started enoxaparin but the treatment was complicated with an hemorrhagic syndrome (grade 2) leading to permanent interruption of ibrutinib. Six patients interrupted ibrutinib due to hematological toxicities and only one restarted at a lower dose.

Fifteen patients developed hematological toxicities other than lymphocytosis: thrombocytopenia in 11 cases (2 grade 4 and 1 grade 3, related to bone marrow infiltration, while the other 8 were judged related to ibrutinib); 2 cases of neutropenia, 1 AML, 1 cytopenia (all grade 4 and judged unrelated to ibrutinib). Lymphocytosis was present in 14 patients at baseline. Ten out of 14 cases of lymphocytosis persisted until the end of the treatment. On the other hand, 13 (16.9%) subjects developed lymphocytosis during therapy, and it then persisted until the end of treatment. The 80% of hematological toxicities occurred in the first 3 months of therapy.

Main extra-hematological toxicities were diarrhea (9.4%) and lung infections (9.0%) which all lead to early drug discontinuation. Overall, 4 (5.2%) atrial fibrillations and 3 (3.9%) hemorrhagic syndromes occurred. The 88% of extra-hematological toxicities occurred in the first 3 months of therapy.

## DISCUSSION

Outcomes are poor for patients with MCL who relapse after initial therapy. There are currently five agents licensed for the treatment of rrMCL: bortezomib (only in USA), temsirolimus (only in Europe), lenalidomide, acalabrutinib (since October 2017, only in USA) and ibrutinib. Nevertheless, there is no standard of care at present in this setting and a definitive MCL treatment algorithm is yet be established [7]. Ibrutinib seems to be one of the more active single agent in refractory/relapsed disease also in the real life setting, but direct comparisons between the four drugs have been never performed in either clinical trials or retrspectice experiences [5, 8].

In our multicenter retrospective study, we evaluated rrMCL patients who were treated with single agent ibrutinib in everyday clinical practice; the most part of the 77 patients were heavily pretreated and at least 50% were primary refractory. Our analysis reported a CR rate of 18.2% that was similar to those observed in clinical trials investigating similar MCL populations treated with ibrutinib as single agent; on the contrary, the ORR rate observed was only 36.4%, rather inferior if compared with data already published [5, 8]. One cause may be the wrong interpretation of lymphocytosis. Approximately a third of patients with MCL develops lymphocytosis during ibrutinib therapy which typically peaks 4 weeks after initiation before slowly declining over subsequent cycles. This transient lymphocytosis does not reflect resistance or disease progression and can be regarded as part of a response to treatment in the context of diminishing tissue bulk elsewhere [9]. In the present work, 14 patients at baseline presented with lymphocytosis and 10 had persistent lymphocytosis until the end of treatment. Seventeen percent developed lymphocytosis during treatment with ibrutinib and it was present at end of treatment too. Physicians wrongly considered lymphocytosis as progression of disease and stopped the treatment. Isolated lymphocytosis should not lead to a diagnosis of disease progression in ibrutinib-treated subjects as lymphocytosis is not to be considered an AE *per se* but reflects the pharmacodynamics of the drug [9].

Regarding survivals functions, our real life data compared with the pooled data from clinical studies, show similar median PFS (12.9 *vs* 12.8 months, respectively) while the median OS appears inferior in our series (16.0 *vs* 25.0 months) [2, 5, 10, 11]. On the contrary, our median DoR was not reached in comparison with 18.6 months of the pooled data report. Of note, 11 (14.2%) patients are in still CR with a median DoR of 36 months at the latest available follow up.

Failure after ibrutinib is an urgent medical need: multiple studies have shown that MCL patients demonstrate a poor outcome in this case [12, 13]. In our report, patients who failed ibrutinib therapy underwent further treatment: 5 out of the 7 patients who underwent ibrutinib rechallenge acquired resistance. This is a common issue which may presumably be solved at least partially with combination or right sequencing therapy [13, 14]. Lenalidomide could be an effective opportunity even if in our study the patients (only five) who underwent lenalidomide after ibrutinib failed to achieve response [15, 16]. On the other hand, patients who consolidated response with SCT had better outcomes.

It is confirmed that ibrutinib has a favorable safety profile, with mild and generally transient side effects. In clinical trial and real life experience, atrial fibrillation (5–8%) and bleeding (3–5%) have emerged as the most challenging safety issues and physicians are inclined to suspend the drug in some instances so as not to make mistakes in the management of these AEs. As a result, patients stop benefiting from a life-saving drug. Recently, practical guidelines for the management of ibrutinib in the real life have been published, focusing on atrial fibrillation and bleeding: this helps physicians to better manage these particular situations allowing responder patients to continue therapy with ibrutinib [17].

To note that the modest side effect profile of ibrutinib candidates this drug to potential combination therapies.

Despite the known potential bias of all observational studies, the present report on the real life experience provides an important contribution to medical knowledge: we have learnt in fact that ibrutinib treatment is effective and well tolerated also in everyday clinical practice. Long-term outcomes and new structured clinical trials are awaited.

## PATIENTS AND METHODS

An observational, non-interventional, multicenter, retrospective observational study was conducted to assess effectiveness of ibrutinib and to analyze outcomes and toxicity data of patients managed in a non-trial setting. The study was approved by our institutional board (Azienda Ospedaliera-Universitaria di Bologna, Policlinico S.Orsola-Malpighi, coordinating Center) and by all involved Ethical Committees and registered in the Italian Registry of Observational Studies. All participants gave written informed consent in accordance with the Declaration of Helsinki.

A shared database was used after the approval of all the authors and variables were strictly defined to avoid bias in reporting data. From July 2014 to January 2015, a total of 29 Italian centers utilized ibrutinib according to the NPP in 77 patients with rrMCL. Centers were consecutively involved to avoid selection bias. Data were collected by local investigators but were monitored and centrally reviewed.

The primary endpoints of the study were the ORR and the CR rate; secondary endpoints were the OS, PFS, disease-free survival (DFS), DoR, and the incidence, severity, and type of any AE occurring during and near after treatment.

Response was assessed by imaging using the International Working Group revised response criteria for malignant lymphoma [18].

Safety and tolerability were evaluated by classifying AEs according to NCI CTCAE version 4.0. OS was defined as the time from initiation of therapy to death from any cause and was censored at the date of last available follow up. PFS was measured from initiation of therapy to progression, relapse, or death from any cause and was censored at the date of last available follow up. DFS was calculated for CR patients from the first documentation of response to the date of relapse or death due to lymphoma or acute toxicity of treatment. DoR was calculated from the first objective tumor response (CR or PR) to first documentation of progression or death [18].

Demographics and patients’ characteristics were summarized by descriptive statistics.

Survival functions were estimated by using the Kaplan–Meier method and were compared using log-rank test.

Statistical analyses were performed with Stata 11 (StataCorp LP, TX) and *p* values were set at 0.05.

## Abbreviations

AE: adverse event
alloSCT: allogeneic stem cell transplant
AML: acute myeloid leukemia
ASCT: autologous stem cell transplant
BTK: Bruton's tyrosine kinase
CCR: continuous complete response
CR: complete response
DFS: disease free survival
DoR: duration of response
ECOG: Eastern Cooperative Oncology Group
MCL: mantle cell lymphoma
NPP: named patient program
ORR: objective response rate
OS: overall survival
PD: progression of disease
PR: partial response
PFS: progression free survival
rr: relapsed/refractory
SD: stable disease
